# Environmental Determinants of Asthma among School Children Aged 13–14 in and around Polokwane, Limpopo Province, South Africa

**DOI:** 10.3390/ijerph6092354

**Published:** 2009-09-03

**Authors:** Kidi Rose Maluleke, Zeleke Worku

**Affiliations:** 1 Research and Development, South African Social Security Agency, PO Box 54684, Wierdapark 0149, South Africa; 2 Department of Statistics, University of South Africa, Pretoria 0002, South Africa; E-Mail:workuzb@unisa.ac.za

**Keywords:** asthma, cough, smoke, flush toilet, odds ratio, multi-level analysis

## Abstract

Asthma has become a public health issue since the 1960’s. Factors associated with asthma are environmental and genetic. This study is based on a random sample of 742 students aged 13–14 attending various schools at Polokwane, in the Province of Limpopo in South Africa. Survey logistic regression and multi-level analyses were used for data analysis. The study identifies three key determinants of asthma at the district, school and individual levels. The study shows that persistent cough (OR = 4.01), exposure to smoke at the household level (OR = 2.39) and lack of access to flush toileta at the household level (OR = 1.89) are key predictors of asthma in children. Variability at the level of districts accounts for 46% of total variance. Variability at the level of schools accounts for 33% of total variance.

## 1. Introduction and Study Background

The Province of Limpopo is one of the poorest and least developed provinces of South Africa that is characterized by poverty among black South Africans and poor infrastructure. School-going children in Limpopo are widely affected by asthma, a disease that is closely associated with indoor pollution, wheezing, use of paraffin and coal, altitude, wind speed, amount of rainfall, humidity, and low socio-economic status. Children with asthma are characterized by breathing difficulties. Children are more at risk of the disease because of their level of physiological development. Recognized biological susceptibility of children has prompted numerous studies in pediatric asthma. Results obtained from the International Study on Asthma and Allergies in Childhood (ISAAC) study that was conducted in Polokwane have shown that the use of paraffin and coal for cooking and indoor heating at households adversely affects 13 to 14 year old children in the district, and that children with poor socio-economic status are particularly vulnerable [1].

Causes of asthma have been studied for a long time. The three perspectives on the origin of this disease are genetic predisposition, environmental factors and their synergistic action. The latter perspective, adopted in this study, is more plausible because it explains why people who are evenly exposed to environmental toxicants do not equally develop asthma [2]. For this particular study, data was gathered from school children aged 13 to 14 in Polokwane based on several socio-demographic, environmental and health-related variables. The aims of this study were to identify key environmental factors that cause asthma among school children aged 13 to 14, and to determine the extent to which the distribution of asthma varies by district and school.

Asthma in children has increased worldwide in both prevalence and severity in the past two decades or so. These increases have prompted volumes of studies to identify risk factors and the distribution of such risk factors in countries globally. The ISAAC studies using standardized methodology are among the many scientific efforts to search for answers. Notwithstanding these efforts, it is not yet clear as to what causes the disease. Several groups of factors were identified. These can be classified into environmental, life style (diets, use of antibiotics, early weaning from breast milk, plastic toys), socio-economic and genetics. Environmental factors, genetic susceptibility, and diets are considered to be the leading causes of asthma and research in these groups of factors has progressed considerably. Socio-economic status (SES) as a causal factor is also entering the scientific literature [3]. But difficulties arise when using SES to explain asthma: SES is a socio-economic concept and biologically it cannot cause asthma; it is defined and measured differently in different contexts such that findings cannot be generalised from one context to another. Inconsistency in findings of studies relating asthma and SES have reflect this difficulty. At this point in time there is no conclusive evidence as to the relationship of asthma and SES. In this study SES is found useful because it signifies the degree to which participants are exposed to risk factors due to poverty or affluence [4].

Adding SES to the list of risk factors makes it possible for the study to contribute directly to millennium development goals of education, health, and indirectly to alleviation of poverty, a condition that has a cause-effect and effect-cause relationship with ill health. Lack of knowledge (ignorance) is correlated with poverty. If this study culminates in an asthma education program for parents or for school children, a correlate of poverty (namely ignorance about asthma) will be addressed. According to Air Pollution Network in Africa (APINA) major polluting sources in SA are mining, energy generation (petrochemicals, electricity generation from coal) and road transportation. Poverty among rural black South Africans is extensive. Ten percent (10%) of the population of South Africa is poor [5]. Even though details of how this poverty is defined and measured were not cited by this source, prevalence of 10% in a population of five million is still substantial. Numerous studies have linked asthma to low socio-economic status (SES). A few examples are studies conducted by [6–11].

Poverty is associated with the occurrence of asthma and its severity. Poverty exposes people to poorer environments that are fraught with asthma risk factors; it makes people incapable of recognizing the disease; it prevents people from having access to adequate medical and health care facilities; it causes people to be unable to manage the disease [12]. Scientific evidence does point to the impact South African country conditions on asthma morbidity and mortality. In the study conducted by Corvalan *et al.* high mortality among children was attributed to asthma [8].

Terblanche *et al.* [13] carried out a study to monitor exposure and prevalence of respiratory illnesses in the Vaal Triangle in South Africa in 1990 to 1992. This area has high concentrations of air pollution emitted from the SASOL petrochemical industry. The exposure monitor study showed high concentrations of TSP (total suspended particles), higher than what is recommended by the World Health Organization. In the other arm of the study, namely, occurrence of respiratory health among children aged 8–12 years, they found the prevalence of lower respiratory infections to be higher among children from non-electrified areas in contrast with children from completely electrified areas (odds ratio 21.9); among children from partially electrified areas in contrast with completely electrified areas (odds ratio 2.3). These studies further indicate the prevalence of air pollution in SA and its effects on respiratory health.

### Objective of the study

The objective of the study is to identify key predictors of asthma in children aged 13 to 14 years living in the Province of Polokwane, South Africa. The study aims to find out the extent to which various socio-economic factors contribute for asthma among school going children in Polokwane.

### List of study variables

Data was gathered from n = 742 pupils aged 13 to 14 years on variables such as age, gender, duration of stay in the environment, use of paraffin, use of coal, household income, level of education of parents, cough, tobacco smoke, diet, pets, weather condition, socio-economic status, access to piped water, ownership of flush toilet, type of household, access to electricity, sanitation, etc.

## 2. Materials and Methods

### Study setting

This study was conducted in an ecological environment that favors occurrence of asthma. Polokwane is characterized by the presence of air pollutants in the ambient air, tropical climate (characterized by warmth, humidity and winds), and low socio-economic conditions in which access to electricity, piped water, proper sanitation and poor environmental sanitation often expose children from poor households to asthma agents.

Silicon smelting, industrial activity, automobile emissions, and smoke from neighborhood—wide use of biomass fuel, are the principal sources of air pollution in the area. The Environmental Health Division of Polokwane Municipality produced the data for suspended particles (soot, smoke, dust and liquid droplets) based on measurements taken monthly over a period of four years (2002–2005) from three stations: Allendale, Burger Centrum and Seshego. Monthly readings were made on average concentration levels of pollutants in ug/m^3^. According to Maritz, pollution figures shown in Table 1 are in excess of levels recommended by the US Environmental Protection Agency [1].

### Geography and weather

Several winds blow across the study area. Tables 2 and 3 below show data on average wind speeds per seconds. Wind speeds are moderately high. Readings taken daily at 8 h 00 and at 14 h 00 for a period of four years show wide variability in wind speeds during the course of each day and each month over that period. Winds mobilize toxic and bioorganic pollutants in their directions. This implies that the degree of exposure to contaminated air differs from district to district in the study area depending upon location in relation to wind speeds and directions of winds.

The Polokwane area is humid. A trend analysis shows high monthly percentages in a period of four years (2002–2005). The highest being 84.2 % in June 2003 and the lowest, 54.4% in September 2005.

Geographic factors, low altitude (1,226 m), position along the latitude of 23.8700 and proximity to the Tropic of Capricorn cause warm climate in Polokwane. Table 5 below gives details. In 2002 monthly average temperatures ranged between 20.3 °C (June) and 29.3 °C (January). Comparative figures for 2003 were 20.7 °C (June) and 30.7 °C (February) suggesting persistent warmer weather. High levels of pollution concentrations and weather combine synergistically to increase the risk of asthma in the area.

### Electrification rates

**Figure 1.**
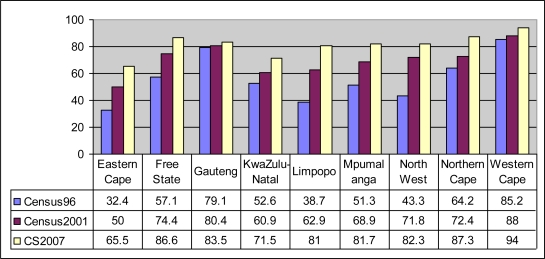
Electrification proportions for South Africa according to Census 2001 and Community Survey 2007.

According to the South African Census of 1996, 57% of South African households had access to electricity. The figure increased to 80% countrywide in 2007 according to the South African Community Survey of 2007 [5]. Figure 1 shows that electrification rates vary significantly by province in South Africa. According to Census 1996, Limpopo Province had only 38.7% electrified households. Based on results [5] obtained from the Community Survey of 2007, there was a sharp increase in electrification between 2001 (62.9%) and 2007 (81%).

In cases where electrification rates are less than 100%, cooking gas, wood, coal, and paraffin become alternative forms of energy. Cooking gas contains methane, nitrogen dioxide, nitric oxide, and cooking fumes. Wood and paraffin produce smoke and soot. These substances are highly inspirable particulate matter that lodges deeper into lungs. Evidence has shown positive influence of these substances on asthma [14].

Presumably, the age cohort of 13 to 14 years may have suffered indoor and outdoor toxic exposure prior to electrification. If this happened in early life before biological development was complete, their respiratory health could have been compromised. This could explain the high asthma prevalence (28%) observed within this sample.

### Sample size of study and sampling technique

The sample size of study was determined using nQuery Advisor version 4 (sample size for single proportions). Figures required for sample size calculations were obtained from Statistics South Africa and the Limpopo Provincial Department of Education. It was assumed that the prevalence of asthma among children with ages 13 to 14 years varied from 11.6% to 17.9%. The total number of children with ages between 13 and 14 in Limpopo is 292, 000. Using a 0.05% level of significance and 99% power, the adjusted sample size of study became 742 children.

Hence, data was gathered from a random sample of 742 school children aged 13 to 14 years who were enrolled at the various schools in Polokwane. A multi-stage cluster sampling technique was used for selecting eligible children from the population of study. First, eligible educational districts in Polokwane were selected on the basis of convenience. Secondly, eligible schools within the selected districts were selected based on maximum variability, convenience and relevance to the study. Next, eligible classrooms within eligible schools were selected. Finally, eligible students were selected from the various eligible classrooms. At each classroom, children who met the criteria for inclusion into the study were selected. The selection of children was done based on PPS (probability proportional to size). The children in the sample were mostly in Grades 7 and 8 (Forms 1 and 2).

### Calculation of sampling weights

Sampling weights were calculated using standard procedures recommended for multi-level health-related surveys. Schools were used as Primary Sampling Units (PSUs). Districts were used as strata. Varying numbers of PSUs (schools) were selected from the various strata (districts).

Stage 1 (school level) selection probability = $P_{1}\;=\;\frac{n_{i}m_{i}}{M}$ where *i* = 1, 2.
  *n_i_* = Number of schools (PSUs) selected from district (stratum)
  *m_i_*= Estimated number of students in school *i* where i = 1, 5
  M = Total number of students in all schools in that particular stratum
Stage 2 (class room level) selection probability = *P*_2_
  $$P_{2}=\;\frac{\text{number}\;\text{of}\;\text{classes}\;\text{selected}}{\text{actual}\;\text{number}\;\text{of}\;\text{classes}\;\text{in}\;\text{school}}$$
Stage 3 (student level) selection probability = *P*_3_
  $$P_{3}\;=\;\frac{\text{number}\;\text{of}\;\text{interviewed}\;\text{students}\;\text{in}\;\text{selected}\;\text{class}}{\text{total}\;\text{number}\;\text{of}\;\text{students}\;\text{in}\;\text{selected}\;\text{class}}$$
Overall selection probability = P = *P*_1_×*P*_2_×*P*_3_
  Sampling weight = $W\;=\;\frac{1}{P}$

Adjustment is done for non-response at the classroom level. The total sample size of study was equal to n = 742 youth with ages between 13 and 14 years. Sampling weights were allocated to each child in the study based on the figures shown in Table 3. The weights were subsequently used for data analysis.

## 3. Data Collection

Before data was collected, the purpose of the study was also explained to each child and parent. Permission was granted for data collection by the Limpopo Provincial Department of Education as well as the various schools that were selected for the study. Parental consent was obtained for each child who took part in the study with help from the various schools. An explanation was given to both children and parents about the purpose and benefit of the study. Trained teachers at the various schools conducted interviews with the children selected for the study. Questionnaires completed by children were collected by interviewers at the various schools, serially numbered and captured. Data validation and verification was done in collaboration with interviewers at the various schools prior to data analysis.

## 4. Data analysis

The outcome variable of study is the presence or absence of asthma in children. The objective of data analysis was to identify key predictors of asthma, and to assess the degree of variability of asthma at the level of districts and schools. To this end, the following statistical methods of data analysis were used: frequency tables for discrete variables of study, summary statistics for continuous variables of study, Pearson’s chi-square tests of association, survey binary logistic regression analysis and multi-level analysis. Odds ratios estimated from logistic regression analysis were used as an epidemiological measure of effect. Multilevel analysis was used for quantifying the variability of asthma at the levels of districts and schools. Data entry and analysis were performed in the statistical package STATA version 10 [15].

## 5. Findings

### Frequency proportions

Fifty six percent (56%) of 742 children in the study were female and black. Sixty five percent (65%) were aged 14 years. Seventy four percent (74%) were from villages, followed by 23.45% from townships, the remainders were from Polokwane city centre and suburbs. The highest proportion (62%) came from Mankweng district.

### Duration of stay in the environment

Table 7 shows figures on duration of stay in the current environment. Prolonged stay is positively and strongly associated with the occurrence of asthma, thereby suggesting that prolonged exposure to a toxic environment has the potential for causing asthma. The majority lived in the area since birth. Table 7 below gives details.

### Seasonal experience of chest problems

One hundred and twenty-six (126) or 16.9% experienced symptoms in late winter (August) and early springs (September) and 69.1% had no such experience. The rest of them could not recall details. Those with asthma stated that symptoms worsened in dusty weather. But dusty weather did not predict asthma in this study [Odds Ratio = 1.13; C.I. = (0.803, 1.607; P = 0.469)]. Absence of published scientific evidence of the relationship of asthma and dusty conditions from other geographic contexts may hinder any causal inferences.

Prevalence of asthma in the sample was 27.63 %. This is more than what was previously observed by Maritz in the same area [1]. The highest prevalence (82%) was in Maune District, the lowest in Mankweng district with 17%. Among females, asthma prevalence was 16.85%. The female-male asthma odds ratio was 1.32 meaning females were 1.3 times more likely to have asthma than males. Differences in perspectives by various theorists on susceptibility of males vs. females and vice versa, make it difficult to interpret this finding within the context of causation.

Prevalence among the 14 year-old group was 29%, and 25% among the 13 year-olds. The odds of asthma among children aged 14 to those aged 13 years was 0.84 meaning older age was protective against asthma. This finding supports a theory of vulnerability of small children to asthma due to incomplete biological development as suggested by Trasande and Thurston [16] as well as Illig and Haldeos [17].

To test whether asthma was associated with the exposures, Pearson chi-square tests of association at the 5% level of significance were run with all variables of the study. Table 8 gives the results. Presence of a smoker within a home p = 0.039, absence of a flush toilet p = 0.022, smoke in the environment p = 0.014 and cough p = 0.000 were significantly associated with asthma.

Table 9 shows results estimated from survey binary logistic regression analysis, a procedure in which the presence or absence of asthma was regressed on nine key predictors of asthma. At the 5% level of significance, influential predictor variables that affect the occurrence of asthma in children are characterized by odds ratios that significantly differ from 1, P-values that are smaller than 0.05 and 95% confidence intervals of odds ratios that do not contain 1. Accordingly, three of the nine variables used for binary logistic regression analysis were significant at the 5% level of significance.

A review of the literature shows that variables such as gender, place of residence and exposure to allergenic diets are potential confounding variables [18]. As a result, the odds ratios estimated from survey logistic regression analysis were adjusted for gender, place of residence and exposure to allergenic diets. Adjusted and unadjusted odds ratios did not differ much, thereby showing that none of the three variables used for adjustment was a confounding variable.

The adjusted odds ratio of the variable “Persistent cough” is 4.01, with a 95% confidence interval of (1.78, 8.09) and a P-value of 0.000. This shows that a child who experiences persistent cough is 4.01 times as likely to develop asthma in comparison with another child who does not experience persistent cough.

The adjusted odds ratio of the variable “Smoke in the environment” is 2.39, with a 95% confidence interval of (1.34, 4.98) and a P-value of 0.000. This shows that a child who is exposed to smoke in the environment is 2.39 times as likely to develop asthma in comparison with a child who is not exposed.

The adjusted odds ratio of the variable “No flush toilet at home” is 1.89, with a 95% confidence interval of (1.01, 5.43) and a P-value of 0.016. This shows that a child living in a household with no flush toilet is 1.88 times as likely to have asthma in comparison with a child who lives in a household where there is a flush toilet.

The above findings show that the key predictors of asthma are persistent cough, living in a smoky environment and having no flush toilet, in a decreasing order of strength.

### Goodness-of-fit tests

Goodness-of-fit tests were done to assess adequacy of the fitted model. The classification table shows that the overall percentage of correct classification is 84.66%, which is fairly high. The fitted logistic regression model is moderately sensitive (56.34%) and highly specific (93.11%). The fitted model predicts pupils with no asthma very well (highly reliable specificity). However, it does not predict pupils with asthma very well. The P-value obtained from the likelihood ratio test is 0.0000 < 0.05. This shows that the variables constituting the fitted logistic regression model are jointly efficient in explaining variability in asthma. Figure 1 below shows the magnitude of area under the Receiver Operating Characteristic (ROC) curve. The magnitude of area that lies under the ROC curve is a measure of explained variation (93.02%).

The large area under the ROC curve in Figure 1 shows that the fitted model accounts for variation in the outcome variable of study fairly well.

### Results from multi-level analysis

The study involves five districts, 10 schools and a variable number of pupils per school. The outcome variable of study denotes the presence or absence of asthma in the child (1, 0). It is generally believed that children who come from the same districts, schools and classrooms are similar with regards to susceptibility to environmental and genetic asthma-causing exposures. Multi–level analysis enables us to assess the degree of variability among districts and schools [19]. That is, does it matter which district or school a child comes from? Such questions are answered adequately using multi-level analysis. A 3-level analysis was done using students as Level 1, schools as Level 2, and districts as Level 3. Three key predictors of asthma were used for multi-level analysis. These three predictor variables were persistent cough (1, 0), exposure to smoke in the environment (1, 0), and ownership of flush toilet at home (1, 0).

Table 10 shows the number of asthmatic children by district. Based on estimates obtained from multi-level analysis, the intra-class-correlation (ICC) for districts is equal to 46%. Hence, variability at the level of districts accounts for 46% of total variance. The intra-class-correlation (ICC) for schools is equal to 33%. Hence, variability at the level of schools accounts for 33% of total variance. School level and district level variations jointly account for 79% of total variance. The effect of schools accounts for 33% of total variation.

When school and district were combined, district level factors explained 46% of the variation, and school level factors explained 33% of the variation. Similarity of students within the same school and districts accounted for 79% of the variation. Similarity of schools within districts accounted for 58% and similarity of pupils within the same school accounted for 33%.

## 6. Discussion of Major Findings

The prevalence of asthma in the sample is 27.63%, suggesting that since the study by Maritz [1], interventions to mitigate environmental factors of asthma described in this study have not worked. This is supported by the finding in this study that polluted air in the living area is a strong predictor of asthma. Genetic predisposition did not significantly predict asthma. This observation is not consistent with the theoretical premise of this study that genetically predisposed individuals develop asthma at the point of interface with environmental risk factors.

Findings on sex and asthma are not easy to assess due to diverse views among scientists on its role in asthma development. However in this study females were found to be more at risk than males (odds ratio 1.32 p = 0.096, C.I. 0.951–1.83), although the finding is not statistically significant. This finding seems to support the claim that male children are more at risk as suggested in Harwood and Fergusson [20].

Higher prevalence of asthma in the younger age group as compared to the older age group (OR 0.84, C.I. 596 −1.19, p = 0.323) shows that older age was protective of asthma. This finding supports the theory of younger children’s vulnerability to adverse health effects of air pollutants as suggested by Illig and Haldeos [17], and that of incomplete physiological development of children as in Trasande and Thurston [16].

Genetic predisposition measured in this study as family presence of allergies and family presence of asthma, did not significantly predict asthma among the 13% who indicated family history of asthma and 39% who indicated family history of allergies. This observation is not consistent with existing theory of genes and asthma as proposed by Paulo [21].

The underlying premise of this study is that effects of air pollutants are dependent upon ones genetic and socio-economic vulnerability. If this premise held, we would observe some strong correlations between asthma/allergies and ownership of flush toilets. In most Sub-Saharan African countries such as South Africa, ownership of flush toilets is a measure of socio-economic status.

The correlation between family history of asthma and exposure to ETS is small and negative (−0.06); and between family history of allergies and exposure to ETS (0.05). Family history of asthma was insignificantly and negatively correlated with living in an environment of smoke (−0.06). Family history of allergies and living in an environment of smoke were also negatively associated (−0.10).

Persistent cough is a strong predictor of asthma. The odds ratio (4.01) and P-value (0.000) show that cough is a key predictor of asthma in children aged 13 to 14 years of age at Polokwane. This evidence supports the emerging theory of rhinoviruses being the connecting link between asthma, common colds and cough as suggested by Friedlander and Busse [22]. This is a unique observation and opens new areas of research.

Weather has been associated with respiratory illnesses [23–26]. In this study dust (as weather condition) did not predict asthma (OR 1.13, p = 0.469) although 18% of subjects indicated experience of asthma symptoms during dusty days. Absence of scientific evidence on health effects of dust on asthma makes it difficult to interpret and infer causation from this finding.

Available evidence relating to meteorological effects (excluding dust) on asthma are on wetness and storms, humidity and dryness, low humidity and cold winters as opposed to milder climate, high and low humidity, cold, heat, fog, wind, rain, heat waves and stronger inversions [27]. This may be because most weather-related asthma studies were carried out in environments where dusty conditions are rare.

Asthma episodes are often seasonal. Almost 17% asthmatics indicated experience of chest problems during late winter (August) and early spring (September). This coincides with a pollen season in South Africa.

Children with persistent cough are 4 times as likely to have asthma in comparison with those who do not have persistent cough [OR 4.01, P = 0.0000, C.I. 1.78–8.09]. Intervention programmes should be designed with a view to alleviate the poor health condition of children suffering from persistent cough.

Smoke in the living environment contributes to unclean ambient air and is causally associated with asthma. It has been suggested that outdoor polluted air is equally effective even if home indoor air is clean [28]. This means students who live in a polluted environment are equally at risk even if they are themselves are not exposed to polluted indoor air. The impact of polluted outdoor air is evident in the odds ratio of 2.39 and P-value of 0.000. This shows that children who live in smoky environments are 2.39 times more at risk of asthma. Given that there is no 100% electrification in the study area, use of unclean fuel is inevitable. This contributes to polluted ambient air in Polokwane areas, suggesting policy intervention to mitigate effects of environmental smoke.

Theory and evidence have proved that environmental tobacco smoke (ETS) is a strong predictor of asthma. In this analysis ETS was significantly associated with asthma in the test of association, p value = 0.039. In binary logistic regression ETS was not a significant predictor (p = 0.215) as well as in multi-level analysis.

In this study older age was protective of asthma. The odds ratio of 14 year olds as against 13 year olds was 0 .79 (C.I. 1.54–1.20). This finding supports the theory of children’s biological vulnerability to asthma agents as suggested by researchers such as Trasande and Thurston [16] as well as Illig and Haldeos [17].

Children with asthma are characterized by lack of access to flush toilets. This finding shows that ownership of a flush toilet is indeed a key SES indicator, and that unhealthy living conditions are associated with the use of non-healthy forms of sanitation and inefficient methods of waste disposal. In this study, children belonging to households with no flush toilets were almost twice as susceptible to asthma in comparison with those coming from households with flush toilets. In most Sub-Saharan African countries such as South Africa, ownership of flush toilets is a measure of socio-economic status.

In a multi-level analysis, some factors improved their predictability and others lost it.

At the district level, living in an environment with smoke (p = 0.014) and cough (p = 0.0000) remained significant predictors of asthma.

At the school level, living in an environment with smoke (p = 0.007) and cough (p = 0.000) were significant predictors. Family history of asthma was marginally predictive (p = 0.167). Family history as a predictor suggests independence/similarity of background among pupils within a school. This may mean that pupils within a school might be coming from a community where people are mostly blood relatives. This is more plausible given the predominantly rural background of subjects and the known homogeneity of rural folks in many aspects of life.

In this study 46% of the asthma cases are explained by district level factors. School level factors explained 33% of the cases. School and district level factors jointly explained 79% of the cases. Similarity of pupils within same school and district was 79%. Similarity of schools within district was 58%. Similarity of pupils within same school was 33%.

## 7. Conclusions

Findings of this study suggested environmental factors to be important predictors of asthma as compared to genetic factors. This calls for policy intervention in mitigation of environmental causes of diseases. Variations in asthma prevalence within districts, and the fact district-level factors explained more cases further points to the need for policy intervention directed at district-level disease causes. The study also linked asthma and rhinoviruses. This unexplored and interesting area of study needs further development. It also has the potential to improve diagnosis of asthma in medical care settings.

## Figures and Tables

**Figure 2.**
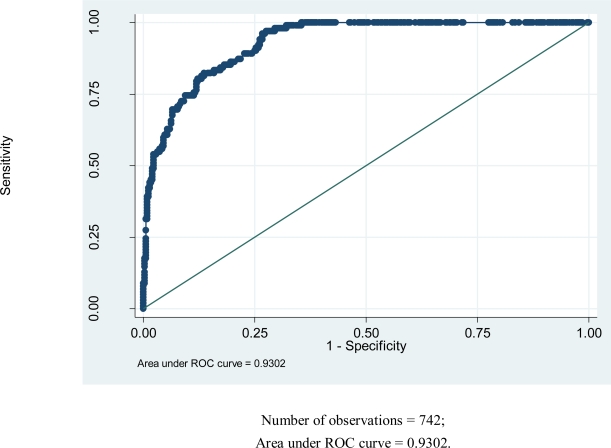
Receiver Operating Characteristic (ROC) plot.

**Table 1.** Air Pollution Average Concentrations for January to December 2002–2005 taken from three monitoring stations, Allandale, Seshego and Polokwane CBD.

**Year**		**Annandale**	**CBD**	**Seshego**

**2002**	**Months**	**AVG S P M**	**AVG SPM**	**AVG SP M**
	Jan	2.7154	1.9213	-
	Feb	1.4244	2.8782	-
	Mar	3.4886	4.1060	-
	April	3.5073	3.8725	-
	May	3.4056	4.2633	-
	June	3.3509	4.6853	-
	July	6.5256	6.8593	-
	Aug	4.2935	4.6613	-
	Sept	3.5040	4.3824	-
	Oct	3.7913	3.3992	-
	Nov	5.0411	3.8229	-
	Dec	6.2011	3.2273	-

**Year**		**Annandale**	**CBD**	**Seshego**

**2003**	**Months**	**AVG SPM**	**AVG SPM**	**AVG S PM**

	Jan	2.7188	3.6574	-
	Feb	2.0231	3.1076	-
	Mar	2.3792	2.8637	-
	April	-	3.5735	-
	May	4.5243	5.5184	-
	June	-	-	-
	July	4.4073	3.7177	6. 8602
	Aug	4.7256	3.9434	6.3443
	Sept	3.5237	4.0485	4.7105
	Oct	2.8550	7.5789	5.9698
	Nov	-	-	-
	Dec	1.5661	5.9572	1.6945

**Year**		**Annandale**	**CBD**	**Seshego**

**2004**	**Months**	**AVG SPM**	**AVG S PM**	**AVG S PM**

	Jan	2.3696	3.4599	2.4658
	Feb	2.1461	-	1.8965
	March	-	-	-
	April	-	-	-
	May	-	-	-
	June	5.0779		10.3994
	July	6.7251	-	11.2531
	August	-	-	-
	September	-	3.7510	5.1734
	October	-	3.6531	4.3243
	November	3.0065	-	3.9486
	December	-	-	-

**2005**	**Months**	**Annandale AVG SPM**	**CBD AVG SPM**	**Seshego AVG S PM**

	Jan	2.6051	3.4637	1.6223
	Feb	2.7362	3.8038	2.2577
	March	2.7719	3.2447	4.0696
	April	2.7032	2.8188	3.4284
	May	-	-	-
	June	-	-	-
	July	-	-	-
	Aug	-	3.7800	5.7322
	Sept	4.1791	5.1030	9.9835
	Oct	2.6863	-	2.7701
	Nov	3.0308	3.2923	2.9464
	Dec	2.9079	2.7624	2.8727

*Source:* Report of Air Pollution 2002–2005 produced by Polokwane Municipality, Environmental Health Divisions; AVG = Average; SPM = Suspended Particulate Matter.

**Table 2.** Polokwane average wind speeds in meters per Seconds measured at 8 h 00 for 2002–2005.

	**Average wind speeds**			

**Months**	**2002**	**2003**	**2004**	**2005**

Jan	3.5	3.6	3.3	3.5
Feb	3.2	3.8	2.4	2.8
March	3.3	3.5	1.5	2.2
April	2.2	3.1	1.3	1.9
May	2.4	4.3	1.6	1.4
June	1.8	3.6	2.0	1.6
July	1.8	3.3	2.1	1.7
August	2.1	3.0	2.2	2.6
Sept	2.7	2.8	2.8	2.2
Oct	3.5	4.1	3.3	4.2
Nov	4.2	3.8	3.6	4.1
Dec	4.6	3.2	3.0	2.8

1 meter per second = 1.944 knots = 3.6 kilometer per hour

Source: South African Weather Services Report of 2006.

**Table 3.** Polokwane average wind speeds in meters per second measured at 14 h 00 for 2002–2005.

	**Average wind speeds**			

**Months**	**2002**	**2003**	**2004**	**2005**

Jan	3.7	4.4	3.0	3.7
Feb	3.6	4.1	3.3	3.3
March	3.6	4.7	3.3	3.2
April	3.5	4.5	3.5	3.5
May	4.2	5.6	3.1	3.6
June	4.1	4.6	3.8	3.7
July	3.4	4.3	3.7	3.3
August	4.9	4.8	4.2	4.9
Sept	4.3	4.4	4.5	4.7
Oct	5.2	5.1	4.8	4.9
Nov	5.1	4.0	3.8	5.1
Dec	4.5	3.6	3.5	3.8

1 meter per second = 1.944 knots = 3.6 kilometer per hour

Source: South African Weather Services Report 2006.

**Table 4.** Monthly and Annual Humidity Percentage at 20 h 00 for the period 2002–2005.

	**% Humidity**			

**Year**	**2002**	**2003**	**2004**	**2005**

January	69.1	60.0	63.3	61.2
February	71.3	62.4	70.0	57.0
March	62.2	60.0	78.0	66.0
April	66.0	60.4	74.0	73.0
May	56.0	57.0	60.3	62.0
June	65.0	72.3	60.4	59.0
July	45.4	51.1	50.1	54.1
August	61.4	37.3	48.0	53.0
September	53.0	43.1	43.1	42.0
October	54.2	49.3	49.0	48.3
November	52.0	58.0	49.0	59.2
December	65.3	55.0	62.1	73.0

Average %	60.075	55.49	58.94	58.98

Source: South African Weather Services Report of 2006.

**Table 5.** Daily Minimum and Maximum Temperatures taken at 08 h 00, 2002–2005.

	**2002**		**2003**		**2004**		**2005**	

**Month**	**Min**	**Max**	**Min**	**Max**	**Min**	**Max**	**Min**	**Max**

January	16.8	29.3	17.6	30.2	18.5	28.4	18.7	29.3
February	16.2	28.5	18.0	30.7	17.8	27.9	17.5	29.6
March	14.8	28.9	15.5	29.6	16.8	25.6	15.7	26.9
April	12.7	27.6	13.2	27.9	13.8	25.4	13.0	24.7
May	8.0	24.4	8.8	24.2	8.4	23.7	9.0	24.2
June	5.3	20.3	6.1	18.9	5.3	19.6	6.7	22.5
July	4.0	22.1	3.3	20.7	4.9	20.2	4.6	27.7
August	8.7	23.8	5.4	23.0	8.4	24.2	9.5	24.4
September	9.3	25.2	9.9	26.1	10.3	25.0	11.6	28.1
October	13.3	27.6	13.8	28.2	14.2	27.3	13.9	28.3
November	13.5	28.5	16.9	28.4	17.1	30.3	16.6	27.7
December	17.0	28.8	18.2	30.1	17.9	28.5	16.1	25.9

Source: South African Weather Services Report of 2006.

**Table 6.** Characteristics of children in study (n = 742).

**Variable**	**Frequency**	**Percentage**

Gender		
Male	326	43.94
Female	416	56.06
Agecat		
14 yrs	486	65.50
13 yrs	256	34.50
Rescat		
Non-village	21	2.83
Village	721	97.17
Smoking		
Non-smoking	540	72.78
Smoking	202	27.22
Tap		
Present	398	53.64
Absent	344	43.36
Toilet		
Present	181	24.39
Absent	561	75.61
Pet		
Present	276	37.20
Absent	466	62.60

Dusty		
Absent	243	32.75
Present	499	62.25
Smoke		
Absent	472	63.61
Present	270	36.39
Milk		
No	65	8.76
Yes	677	91.24
Eggs		
No	73	9.84
Yes	669	90.16
Peanuts		
No	62	8.36
Yes	680	91.64
Fish		
No	33	4.45
Yes	709	95.55
Family1		
Yes	96 646	12.94
No	646	87.06
Family2		
Yes	287	38.68
No	455	61.32
Asthma		
Yes	205	27.63
No	537	72.37
Cough		
Yes	227	30.59
No	515	69.41
Indoor		
Yes	275	37.06
No	467	62.94
SES		
Low	539	72.64
Otherwise	203	27.36
Dietexp		
Yes	479	64.56
No	263	35.44
Toxins		
Yes	46	6.20
No	696	93.80

**Table 7.** Duration of Stay and Asthma Prevalence.

**Duration of Stay**	**Number of Asthma cases**	**Prevalence in %**

Since birth	98	47.80
More than 3 years	64	31.21
1–2 years	18	8.9
9–12 months	11	5.36
Less than 6 months	14	6.83

Total	205	100

**Table 8.** Tests of associations between asthma and predictors.

**Outcome Exposure**	**Chi Sq**	**P value**

**Asthma Gender**	2.7734	0.096

**Age categories**	0.9785	0.323
**Residence**	2.5068	0.113
**Duration**	4.4757	0.345
**Smoking**	4.2609	0.039
**Tapped water**	0.2506	0.617
**Toilet**	5.2567	0.022
**Pet ownership**	0.0019	0.966
**Dusty**	0.5236	0.469
**Smoke**	6.0418	0.014
**Milk**	0.0774	0.781
**Eggs**	0.6092	0.435
**Peanuts**	3.0335	0.082
**Fish**	0.5622	0.453
**Family1**	0.7235	0.395
**Family2**	3.2581	0.071
**SES**	0.2882	0.591
**Dietexp**	3.1484	0.076
**Toxins**	0.8506	0.356
**Cough**	68.0016	0.000

**Table 9.** Estimates from survey binary logistic regression analysis.

**Variable**	**Unadjusted Odds Ratio**	**Adjusted Odds Ratio**	**P-value**	**95% C. I.**

Persistent cough	3.94	4.01	0.000	(1.78, 8.09)
Smoke in the environment	2.36	2.39	0.000	(1.34, 4.98)
No flush toilet at home	1.88	1.89	0.016	(1.01, 5.43)

* Adjustment was done for gender, place of residence and exposure to allergenic diets.

**Table 10.** Prevalence of asthma by district.

**District**	**Asthma Cases**	**Total Sample**	**Prevalence %**

Bahlaloga	15	23	65%
Mankweng	79	454	17%
Maraba	36	104	35%
Maune	16	19	82%
Seshego	59	142	42%

**Total**	**205**	**742**	**27.6**
